# Phylogeographic Structure Reveals Hidden Diversity Patterns in *Tor tambra* (Cyprinidae) Across Thai River Systems

**DOI:** 10.3390/ani16030517

**Published:** 2026-02-06

**Authors:** Vatthanachai Phanklam, Sommai Janekitkarn, Kathathep Seesan, Narongrit Muangmai, Cong Zeng, Prapansak Srisapoome, Kornsorn Srikulnath, Ling Wu, Xiao-Yong Chen, Uthairat Na-Nakorn, Ishwar S. Parhar

**Affiliations:** 1 Department of Fishery Biology, Faculty of Fisheries, Kasetsart University, Bangkok 10900, Thailand; rujfisheries66@gmail.com (V.P.); sommai.j@ku.th (S.J.); kathathep.s@ku.th (K.S.); 2 Biodiversity Center, Kasetsart University (BDCKU), Bangkok 10900, Thailand; 3 School of Oceanography, Shanghai Jiao Tong University, Shanghai 200030, China; congzeng@sjtu.edu.cn; 4 Department of Aquaculture, Faculty of Fisheries, Kasetsart University, Bangkok 10900, Thailand; ffispssp@ku.ac.th (P.S.); ffisurn@ku.ac.th (U.N.-N.); 5 Animal Genomics and Bioresource Research Unit (AGB Research Unit), Faculty of Science, Kasetsart University, Bangkok 10900, Thailand; kornsorn.s@ku.ac.th; 6 Southeast Asia Biodiversity Research Institute, Chinese Academy of Sciences, Yezin 05282, Myanmar; wuling@mail.kiz.ac.cn (L.W.); chenxy@mail.kiz.ac.cn (X.-Y.C.); 7 Yunnan Key Laboratory of Biodiversity and Ecological Conservation of Gaoligong Mountain, Kunming Institute of Zoology, Chinese Academy of Sciences, Kunming 650223, China; 8 Brain Research Institute Monash Sunway (BRIMS), Jeffrey Cheah School of Medicine and Health Science, Monash University Malaysia, Bandar Sunway 47500, Malaysia; 9 Center Initiative for Training International Researchers, University of Toyama, Toyama 930-0194, Japan

**Keywords:** populations, biogeography, phylogeny, distribution, genetic diversity

## Abstract

Understanding patterns of genetic diversity and population structure in freshwater fishes is crucial for evolutionary studies and conservation planning. *Tor tambra* is a common freshwater fish species in Thai waters, yet its genetic diversity and population connectivity remain largely unexplored. This study investigated the phylogeographic structure and genetic diversity of Thai *T. tambra* through phylogenetic analyses and molecular approaches using mitochondrial DNA markers (COI and Cyt*b*). Our analyses revealed four distinct genetic lineages (clades A-D) within Thai *T. tambra*. Lineage C comprised specimens from southern Thailand, Malaysia, Indonesia, and southern China, while lineages A, B, and D were distributed exclusively within Thailand. These Thai-endemic lineages exhibited pronounced geographic structuring across northern and western regions, with lineage A widespread, lineage B confined to northern areas, and lineage D restricted to western localities. The genetic differentiation and distribution patterns of these lineages suggest influence from the limited north–south river system connections and the biogeographic barrier posed by the Isthmus of Kra. Multiple lineages co-occurred at some locations, indicating a complex evolutionary history. These findings provide important implications for understanding the evolutionary processes and biogeographic factors shaping genetic diversity within *T. tambra* across the riverine systems of Thailand.

## Simple Summary

Thai mahseer (*Tor tambra*) populations harbor hidden genetic diversity that has important implications for their conservation and management. By analyzing mitochondrial DNA from fish collected across nine Thai river systems, we discovered four distinct genetic lineages with unique geographic distributions. Southern populations formed a separate group from northern populations, reflecting the influence of the Isthmus of Kra as a biogeographic barrier. Northern and western river systems showed complex patterns with multiple lineages coexisting, suggesting a dynamic evolutionary history shaped by ancient river connections during glacial periods. We also resolved a taxonomic puzzle: specimens from Malaysia and Vietnam, previously identified as different species, are actually misidentified *T. tambra*. These findings highlight the importance of protecting each genetic lineage as a distinct conservation unit and underscore the need for integrated approaches combining molecular and morphological data in mahseer taxonomy.

## 1. Introduction

Freshwater ecosystems are recognized as hotspots of biodiversity and endemism globally, harboring exceptionally high levels of fish diversity [1]. However, freshwater fish are among the most threatened taxonomic groups, facing multiple anthropogenic pressures including habitat loss, overfishing, pollution, invasive species, and climate change [1,2]. Understanding phylogeographic patterns and population genetic structure has become increasingly crucial for informing science-based management and conservation of freshwater fishes [3,4,5]. Particularly, elucidating the spatial distribution of genetic diversity and historical patterns of population connectivity is essential for maintaining ecosystem-scale evolutionary resilience. For species like mahseers that face conservation challenges, this necessitates sustained genetic monitoring within an adaptive conservation management framework to ensure long-term persistence of genetically robust populations [6,7,8].

The complex geological history and topography of Southeast Asia have profoundly shaped the distribution and genetic structure of freshwater organisms. Major geographic features, particularly mountain ranges and the Isthmus of Kra, create significant barriers to gene flow among populations [9,10,11]. The Isthmus of Kra, located at approximately 10° N latitude in southern Thailand, acts as a major biogeographic boundary separating northern and southern river systems and has been recognized as a key driver of phylogeographic breaks in Southeast Asian freshwater fauna. Phylogeographic studies of freshwater fishes in this region have revealed distinct genetic differentiation patterns corresponding to these geographic barriers, with notable genetic breaks between northern and southern populations [12,13]. Within Thai territory, the isolation of river systems by mountain ranges has been identified as a key factor driving population genetic structure in several freshwater species [12,14,15]. The biogeographic history of Southeast Asian freshwater fauna has been further influenced by Pleistocene climatic oscillations and associated sea-level fluctuations. During glacial maxima, sea levels dropped by up to 120 m, exposing the Sunda Shelf and creating extensive palaeodrainage networks, including major palaeorivers that facilitated temporary river system connectivity and faunal exchanges between currently isolated drainages [16,17,18]. Subsequent post-glacial sea-level rises re-established drainage barriers, leading to population isolation and genetic differentiation across multiple river basins [10,16]. These biogeographic patterns provide essential context for understanding the evolution and genetic diversity of Southeast Asian freshwater fishes, including economically important species such as mahseers.

Among the diverse freshwater ichthyofauna of Southeast Asia, mahseers (genus *Tor*) represent an important group of cyprinid fishes distributed across major river systems from India to Indonesia [19,20,21,22]. These fish hold significant ecological and economic value in both aquaculture production and capture fisheries. However, *Tor* populations have experienced severe declines due to habitat fragmentation from dam construction, overexploitation, pollution, and climate change impacts [20,21,23].

In Thailand, *Tor tambra* is widely distributed across various river systems and serves as an excellent model for investigating biogeographic patterns in Southeast Asian freshwater fishes [21,22]. Previous studies have suggested complex patterns of genetic structuring in *T. tambra* populations across Southeast Asia [5,23,24,25,26]. However, while previous studies of Thai *T. tambra* have focused on phylogenetic and genomic analyses [19,27], comprehensive assessments of population connectivity and genetic structure across major river basins within Thai territory remain limited. Additionally, the diverse topography of Thailand, characterized by mountainous regions, extensive river networks, and distinct watershed boundaries, provides an opportunity to examine how historical geological events and contemporary landscape features influence genetic diversity and population structure in freshwater fishes.

This study aims to elucidate the phylogeographic patterns and genetic diversity of *T. tambra* populations across major river basins of Thailand. Using mitochondrial DNA markers, we investigate historical patterns of population connectivity, genetic structure, and the influence of geographic barriers on population differentiation. Understanding these patterns is crucial for developing effective conservation strategies that maintain genetic diversity and evolutionary potential in this economically and ecologically important species.

## 2. Materials and Methods

### 2.1. Sampling

A collection of *Tor tambra* samples was made from nine freshwater localities in Thailand (Table 1 and Table S1). Specimens collected were identified as *T. tambra* based on their key diagnostic characters described in previous studies of [21,26,28], including the presence of a distinctive short median lower lobe, relatively thin lips, and a blunt or rounded head shape. Voucher specimens were deposited at the Faculty of Fisheries, Kasetsart University, Thailand. The specimens were cleaned to remove epiphytes and mud. Right pectoral fins were removed and stored in 100% alcohol at −20 °C for DNA extraction. The remaining samples were preserved as voucher specimens for further morphological observations.

### 2.2. DNA Extraction, PCR, and Sequencing

Total DNA was isolated from fin tissue using the NucleoSpin Tissue kit (Macherey-Nagel, Duren, Germany), following the manufacturer’s protocol, with final elution in 100 μL elution buffer. DNA quality was assessed using NanoDrop spectrophotometry (Thermo Fisher Scientific Inc., Waltham, MA, USA) prior to PCR amplification. Two mitochondrial genes, cytochrome c oxidase subunit I (COI) and cytochrome B (Cyt*b*), were used in this study. The sequence of the COI and Cyt*b* regions was amplified using the previously published primers Fish F1 and Fish R2 [29] and LA-cyp and HA-cyp [30], respectively. PCR was performed using WizPure™ PCR 2X Master (Wizbiosolutions Inc., Gyeonggi-do, Korea) following the manufacturer’s instructions with amplification profiles and procedure according to [29] for COI (initial denaturation 94 °C for 2 min; 35 cycles of 94 °C for 30 s, 54 °C for 40 s, 72 °C for 1 min; final extension 72 °C for 10 min) and [30] for Cyt*b* (initial denaturation 95 °C for 5 min; 35 cycles of 95 °C for 1 min, 50 °C for 1 min, 72 °C for 1.5 min; final extension 72 °C for 7 min). All successful PCR products were cleaned using ExoSAP-IT (USB, Cleveland, OH, USA) and then sequenced commercially (U2Bio Inc., Seoul, Republic of Korea).

### 2.3. Alignment, Phylogenetic Reconstructions, and Haplotype Analysis

All COI and Cyt*b* sequences were checked and edited in Geneious Prime^®^ 2025.0.2 (Biomatters, http://www.geneious.com, accessed on 10 October 2025). Multiple sequence alignment was performed using the MUSCLE algorithm implemented in Geneious Prime and then manually refined. Additional sequences of COI and Cyt*b* of *T. tambra* and related species were retrieved from NCBI and included in our dataset.

Phylogenetic analyses were conducted using maximum likelihood (ML) and Bayesian inference (BI) methods on both individual gene datasets, COI and Cytb, and the concatenated dataset. Both methods were employed to provide complementary assessments of phylogenetic relationships, with ML offering computational efficiency and BI providing probabilistic support for node reliability. ML analyses were performed with the best-fit model (partitioned by gene and codon position) selection implemented in ModelFinder [31]. The nodal support values were estimated using the nonparametric bootstraps with 1000 replicates [32]. For BI analyses, the nucleotide substitution models were selected using Kakusan 4 [33]. BI analyses were conducted by two parallel runs of four Markov chains for two million generations with sampling every 1000 generations. Convergence was assessed by examining effective sample sizes (ESS > 200) and the standard deviation of split frequencies (<0.01). The first 500 trees (25% burn-in) were removed before determining a consensus tree and posterior probabilities (PP). The best-fit partition and model of ML and BI analyses for all datasets were presented in Table S2. Trees were redrawn in FigTree V1.4.4 (http://tree.bio.ed.ac.uk/software/figtree/, accessed on 10 October 2025) and then edited using Adobe Illustrator 2025 (Adobe Systems Incorporated, San Jose, CA, USA).

Genetic distances were calculated using the Kimura 2-parameter (K2P) model with complete deletion of gaps in MEGA 11 [34]. The K2P model was selected as it is widely used for intraspecific genetic distance calculations in fish phylogeography, allowing direct comparison with published studies on *Tor* and other cyprinids. Haplotype networks for both individual and concatenated datasets of COI and Cyt*b* were constructed using statistical parsimony implemented in PopART [35].

## 3. Results

### 3.1. Genetic Variation and Phylogenetic Analyses

**Figure 1:**
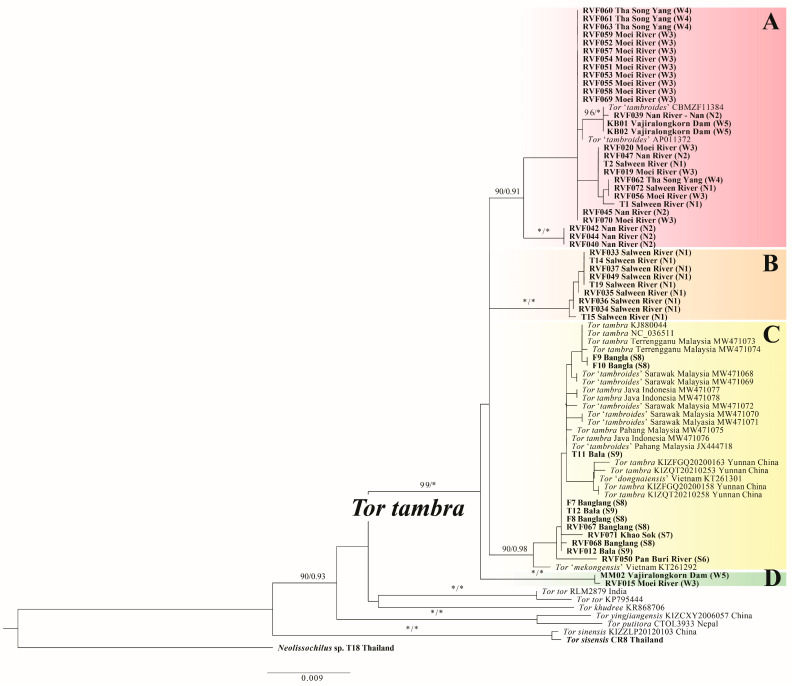
A maximum likelihood phylogenetic tree was constructed based on concatenated COI and Cyt*b* sequences, with sequences generated in this study highlighted in bold. Support values (bootstrap/posterior probabilities) are indicated, with asterisks * denoting 100%/1.00 support; values < 90%/<0.90 are not shown. Specimen details are available in Table S1.

The partial COI (672 bp) and Cyt*b* (1140 bp) fragments were successfully amplified and sequenced from 56 and 50 individuals, respectively, of *Tor tambra* collected from 9 locations across Thailand (Table 1 and Table S1). Of these, 50 individuals yielded high-quality sequences for both markers and were used in concatenated analyses (Table 1). Genetic distance analyses within Thai *T. tambra* samples using the K2P model revealed intraspecific variation ranging from 0.18–2.57% for COI (Table S3) and 0.10–2.44% for Cyt*b* (Table S4). Three samples from Nan Province (RVF040, RVF042, RVF044) showed conflicting phylogenetic placements between individual marker trees, grouping with Clade A in the COI tree (Figure S1) but with Clade B in the Cyt*b* tree (Figure S2); such discordance may reflect incomplete lineage sorting or historical introgression events. However, the combined dataset analysis resolved all three samples in Clade A with strong support (Figure 1, Table S1). The topologies of phylogenetic trees obtained from both maximum likelihood (ML) and Bayesian inference (BI) analyses were generally congruent for the concatenated dataset and individual markers, with minor differences observed only in the placement of samples or weakly supported clades. Given the high congruence between methods and for clarity of presentation, only the ML tree based on the concatenated dataset is presented (Figure 1), with support values from both ML and BI analyses indicated on nodes.

Phylogenetic analyses of individual markers (COI, Figure S1; Cyt*b*, Figure S2) and combined datasets (Figure 1) consistently showed that Thai samples formed a well-supported monophyletic clade with *T. tambra* from Malaysia, Indonesia, and China, as well as specimens previously identified as *T*. “*tambroides*” (AP011372, HM536923, MW471068-MW471072, JX444718), *T.* “*dongnaiensis*” (KT261301), and *T.* “*mekongensis*” (KT261292). All three phylogenetic trees resolved the same four geographic clades (A–D) within *T. tambra* and demonstrated that these specimens are nested within the *T. tambra* clade rather than forming distinct lineages. Genetic distance analyses using an expanded COI dataset (136 sequences, Figure S1, Table S5) revealed that these specimens showed K2P distances of only 0.18–2.19% from Thai *T. tambra*, falling entirely within the intraspecific variation reported above. In contrast, *Tor tambroides* from Indonesia (Sumatra and Java) showed distances of 2.82–4.46%, representing approximately 2.5-fold higher divergence. This genetic discontinuity strongly supports that *T.* “*tambroides*”, *T.* “*dongnaiensis*”, and *T.* “*mekongensis*” specimens within the *T. tambra* clade represent taxonomic misidentifications and are conspecific with *T. tambra*, while *T. tambroides* from Indonesia are recognized as a distinct species. Future morphological reassessment of these specimens would provide additional validation of these molecular findings.

Within the *T. tambra* clade, the combined dataset resolved four well-supported clades (A–D) with distinct geographic distributions (Figure 1, Table S1). Clade A (ML = 90%, BI = 0.91) comprised 33 specimens from northern Thailand (Nan Province, n = 7) and western Thailand (Tak Province, n = 20; Mae Hong Son Province, n = 3; Kanchanaburi Province, n = 3; Table 1). This clade also included three reference specimens identified as *T.* “*tambroides*” (AP011372, HM536923) and *T.* “*dongnaiensis*” (KT261301). Clade B (ML = 100%, BI = 1) consisted exclusively of 9 specimens from the Salween River system in Mae Hong Son Province (northern Thailand), forming a geographically cohesive lineage sister to Clade A. Clade C (ML = 90%, BI = 0.98) showed the broadest geographic distribution with 12 Thai specimens, all from southern Thailand (Yala Province, n = 7; Narathiwat Province, n = 3; Surat Thani Province, n = 1; Prachuap Khiri Khan Province, n = 1), along with reference sequences from multiple countries and seven specimens representing misidentified taxa: six identified as *T.* “*tambroides*” (MW471068-MW471072, JX444718) and one identified as *T.* “*mekongensis*” (KT261292). Clade D formed a small but distinct lineage containing only 2 specimens from western Thailand (Kanchanaburi Province, n = 1; Tak Province, n = 1), representing the most divergent Thai lineage within *T. tambra* and appearing as sister to all other Thai clades.

Pairwise genetic distances among the four *T. tambra* clades are summarized in Table 2. For COI, interclade distances ranged from 1.27% to 2.57%, while Cyt*b* distances ranged from 1.45% to 2.44%. Clade D consistently exhibited the highest divergence from other clades across both markers, particularly in COI comparisons. Additionally, Clades B and C showed the lowest interclade divergence despite their geographic separation between northern and southern Thailand (COI: 1.50%, Cyt*b*: 1.55%). The different ranking patterns of pairwise distances between COI and Cyt*b* (detailed in Table 2) likely reflect the distinct evolutionary dynamics of these mitochondrial markers.

### 3.2. Haplotype Network and Geographic Distribution

**Figure 2:**
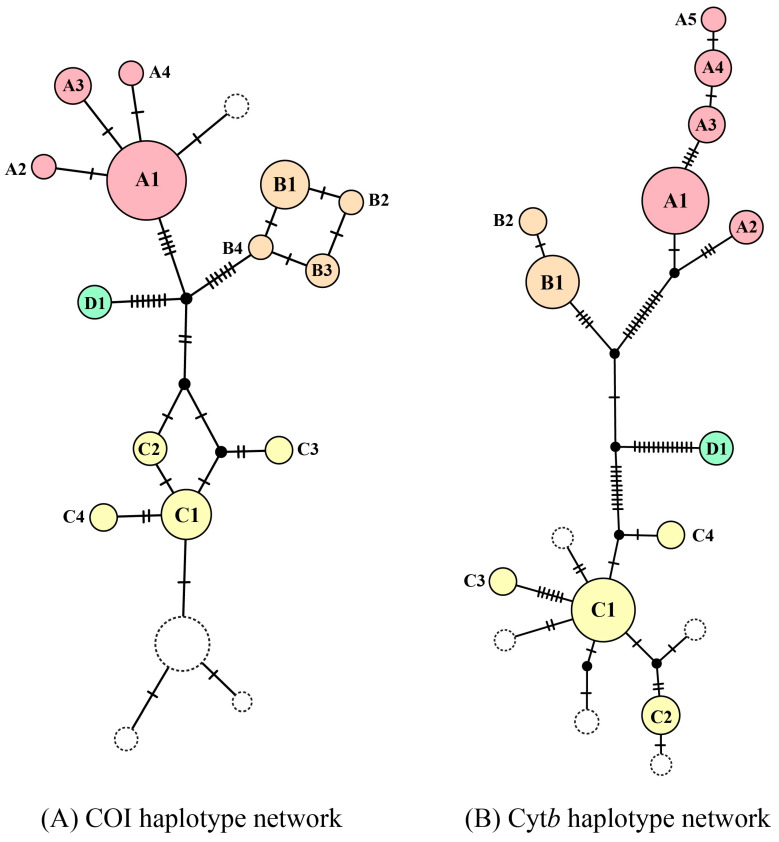
Haplotype networks of cryptic *Tor tambra* species based on mitochondrial gene sequences: (**A**) COI and (**B**) Cyt*b* gene. Each color represents a distinct genetic lineage within the *T. tambra*: clade A (red), clade B (orange), clade C (yellow), and clade D (green). The size of each pie chart is proportional to the frequency of the corresponding haplotype. Solid circles indicate haplotypes identified in this study, while dashed circles represent haplotypes retrieved from GenBank. Small black nodes depict inferred missing or extinct haplotypes connecting the observed haplotypes within the network.

Haplotype network analyses based on COI (56 specimens) and Cyt*b* (50 specimens) revealed 13 haplotypes for COI and 12 haplotypes for Cyt*b* (Table 1, Figure 2). When markers were combined, individual specimens with the same haplotype for one marker but different haplotypes for the other marker produced distinct concatenated haplotypes, resulting in higher haplotype diversity in the combined dataset. Both markers showed congruent clustering patterns, with haplotypes grouped into four distinct haplogroups (A–D) corresponding to the phylogenetic clades (Figure 1, Figures S1 and S2). For individual markers, haplogroup A was widely distributed across northern and western Thailand, haplogroup B was restricted to northern Thailand, haplogroup C was confined to southern Thailand, and haplogroup D was limited to western Thailand (Table 1, Figure 2).

**Figure 3:**
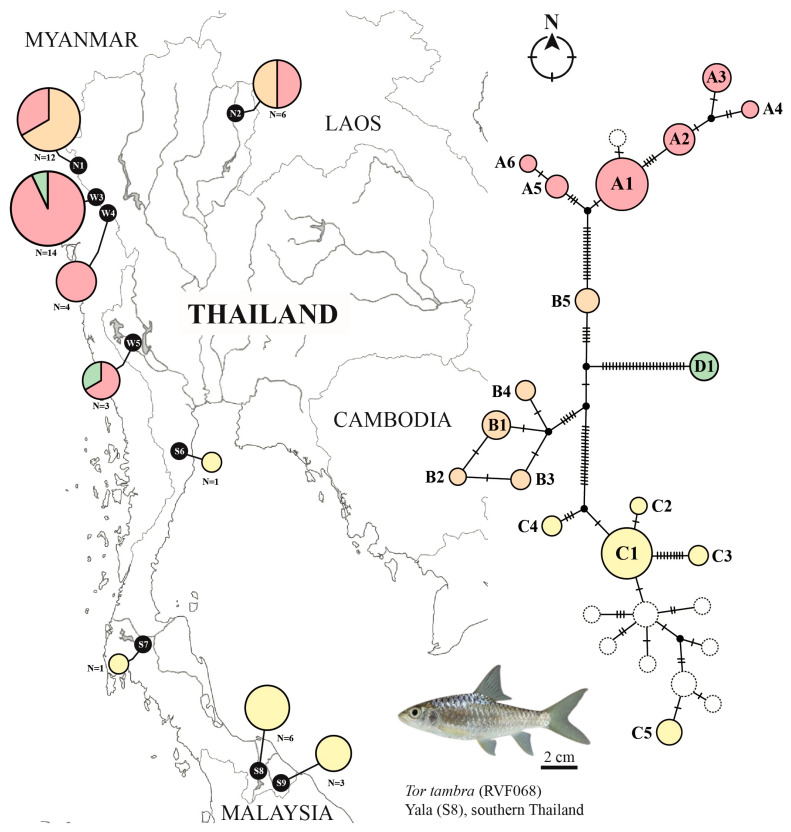
Phylogeographic structure and haplotype network of cryptic *Tor tambra* species across Thailand, based on concatenated sequences of the mitochondrial genes COI and Cyt*b*. Pie charts overlaid on the map indicate the proportional representation of cryptic *T. tambra* populations. Each pie chart is labeled with the respective population code and total sample size (N), as detailed in Table 1. Each color represents a distinct genetic lineage within the *T. tambra*: clade A (red), clade B (orange), clade C (yellow), and clade D (green). The size of each pie chart in the haplotype network is proportional to the frequency of the corresponding haplotype. Solid circles denote haplotypes identified in this study, while dashed circles represent haplotypes obtained from GenBank. Small black nodes illustrate inferred missing or extinct haplotypes connecting observed haplotypes within the network.

Analysis of the concatenated dataset yielded 17 distinct haplotypes (Figure 3, Table 1), which is greater than the number of haplotypes observed for individual markers (COI: 13, Cyt*b*: 12) due to different combinations of COI and Cyt*b* haplotypes within specimens. The concatenated dataset maintained the four-haplogroup structure observed in phylogenetic analyses. Haplogroup A comprised six haplotypes (A1–A6) widely distributed across northern and western Thailand, with A1 found primarily in western Thailand (Tak Province: 13 individuals), A2 and A3 shared between northern (Mae Hong Son and Nan provinces) and western populations (Tak Province), A4 found in northern Thailand (Mae Hong Son Province), A5 restricted to western Thailand (Kanchanaburi Province), and A6 found in northern Thailand (Nan Province). Haplogroup B contained five haplotypes (B1–B5) exclusively distributed in northern Thailand, with B1–B4 found in Mae Hong Son Province and B5 restricted to Nan Province. Haplogroup C encompassed five haplotypes (C1–C5) confined to southern Thailand, where C1 was the most widespread haplotype found in Yala and Narathiwat provinces, while C2–C5 represented province-specific or rare haplotypes. Haplogroup D maintained a single haplotype (D1) exclusively found in western Thailand (Tak and Kanchanaburi provinces).

## 4. Discussion

### 4.1. Genetic Diversity of T. tambra in Thailand

Our genetic analyses based on mitochondrial COI and Cyt*b* markers revealed substantial genetic diversity within *T. tambra* populations across Thailand, with four distinct lineages (A–D). The genetic distances between these lineages (COI: 1.27–2.57%; Cyt*b*: 1.45–2.44%) exceed typical intraspecific variation in other freshwater fishes. In cyprinid fishes, average intraspecific COI divergences typically range from 0.2–1.0%, with a mean of 0.73% across 752 North American freshwater fish species [36]. The relatively high genetic distances observed in some *T. tambra* lineages suggest ongoing genetic differentiation, with some comparisons approaching or slightly exceeding the 2% threshold commonly used as a starting point for species delimitation in fishes [37,38].

Comparative analyses with other *Tor* species reveal consistent patterns of substantial intraspecific variation across Southeast Asia. *Tor putitora* shows COI divergences of 0.8–2.1% across its range [39], *T. douronensis* exhibits genetic distances of 1.2–2.4% between river systems in Sarawak [40], Indian *Tor* species exhibit COI divergences of 0.26–3.0% [39] and *T. tambroides* demonstrates divergences of 1.8–2.5% across Malaysian watersheds [24]. Importantly, genetic distance thresholds for species boundaries vary considerably across fish lineages, with optimized values of 1.5% reported in African Cyprinidae [41] and 2% serving as a heuristic starting point rather than a definitive criterion in Neotropical freshwater fishes [38]. However, it should be noted that threshold-based approaches to species delimitation have inherent limitations, as genetic distances alone cannot account for biological factors such as reproductive isolation, ecological differentiation, or demographic history. The level of genetic differentiation in Thai *T. tambra* populations falls within the range of intraspecific variation documented for the genus *Tor*, suggesting that high intraspecific variation is characteristic of this genus. Therefore, we interpret our four lineages as phylogeographic units rather than cryptic species, a distinction requiring validation through integrative taxonomy combining nuclear markers, morphological analyses, and ecological data. This pattern likely reflects both ecological factors, including restricted movement between river systems, high site fidelity, and specific habitat requirements [20,42], and the complex geological history of Southeast Asia, where repeated sea-level fluctuations and river capture events during the Pleistocene contributed to population isolation and subsequent genetic differentiation [10].

### 4.2. Taxonomic Implications of Misidentified Specimens

Our phylogenetic analyses identified taxonomic inconsistencies within the *Tor tambra* species complex, with ten specimens previously identified as three different species, including *T.* “*tambroides*” from Malaysia, *T.* “*dongnaiensis*” and *T.* “*mekongensis*” from Vietnam, nested within the *T. tambra* clade rather than forming distinct lineages. These specimens showed genetic distances of only 0.18–2.19% from Thai *T. tambra*, falling entirely within the observed intraspecific variation, while *T. tambroides* from Indonesia exhibited substantially higher divergence (2.82–4.46%). This finding has important implications for mahseer taxonomy and conservation across Southeast Asia.

The widespread misidentification of *T. tambra* specimens as distinct species likely stems from the historical reliance on morphological characters that exhibit high plasticity in response to environmental conditions. Mahseer species are notoriously difficult to distinguish morphologically due to overlapping meristic characters and phenotypic variation influenced by habitat differences [19,25,26]. Our molecular evidence strongly supports the need for taxonomic revision of the *T. tambra* species complex, including formal assessment of whether *T.* “*dongnaiensis*” and *T.* “*mekongensis*” represent junior synonyms of *T. tambra*. However, formal nomenclatural actions, including the designation of synonymies, require comprehensive morphological examination and are beyond the scope of this molecular study. This finding nevertheless highlights the importance of integrative taxonomic approaches combining molecular and morphological data for accurate species delimitation in this taxonomically challenging group [20].

From a conservation perspective, the recognition that populations across Thailand, Malaysia, and Vietnam represent a single widespread species rather than multiple distinct species has important management implications. Conservation resources can be better allocated by treating *T. tambra* as a single species with multiple evolutionary significant units (ESUs) based on the four genetic lineages identified in this study, rather than managing misidentified populations as separate species. This approach aligns with modern conservation genetics principles that emphasize the preservation of evolutionary potential and adaptive diversity within species [43].

### 4.3. Phylogeographic Patterns

The distribution of genetic lineages of *T. tambra* across Thailand reveals intriguing biogeographic patterns that reflect the complex geological history of the region. The four deeply divergent haplogroups (A–D) exhibited strikingly different distributions: Haplogroup A was uniquely widespread across northern and western Thailand, Haplogroups B and C were regionally restricted to northern and southern Thailand, respectively, while Haplogroup D appeared as a rare western lineage. The clear north–south differentiation, particularly evident in Haplogroup C, aligns with the Isthmus of Kra (approximately 10°30′ N) as a biogeographic barrier formed during the late Miocene-early Pliocene (~5.5–4.5 Ma) [9,44]. The complete absence of haplotype sharing between southern and northern/western populations underscores the effectiveness of this barrier, maintained by hydrological isolation of the Chao Phraya, Mae Klong, and Tapi River systems during Pleistocene sea-level fluctuations [16]. While geographic barriers provide the primary explanation for this pattern, ecological filtering related to differences in habitat characteristics between northern and southern river systems may also contribute to lineage segregation.

The unique trans-drainage distribution pattern of Haplogroup A suggests historical connectivity between the Salween and Moei River systems, likely facilitated by river capture events during the late Pliocene uplift of the Central Plain (~3 Ma) [45]. These drainage connections were reinforced during Pleistocene glacial periods when lowered sea levels created extensive palaeodrainage networks across the exposed Sunda Shelf and mainland Southeast Asia, enabling faunal dispersal between currently isolated river systems [16,17,18]. The numerical dominance of this haplogroup in western Thailand versus lower northern frequencies suggests either a western refugium or recent demographic expansion. The co-occurrence of multiple haplogroups in northern and western localities (N1, W3-W5) likely reflects either glacial refugia or post-glacial secondary contact along these historical corridors [16]. Similar trans-drainage dispersal has been documented in *Barbonymus schwanenfeldii* [46], indicating river capture was a common phenomenon shaping cyprinid biogeography in mainland Southeast Asia.

In contrast, the exclusive northern distribution of Haplogroup B with fine-scale structuring between Mae Hong Son and Nan River systems reflects the strength of watershed boundaries as dispersal barriers, even between adjacent drainages. The multiple co-occurring haplotypes in Mae Hong Son indicate this region likely served as a Pleistocene refugium, allowing in situ diversification [47,48]. Southern Haplogroup C showed a dominant widespread haplotype alongside rare provincial variants, indicating historical gene flow within the peninsula via ancient Sunda drainage connections during Pleistocene low sea stands [18], followed by recent isolation after post-glacial sea-level rises. This pattern parallels those in *Hampala macrolepidota* [49] and *Channa striata* [50].

The enigmatic Haplogroup D, represented by a single deeply divergent haplotype currently documented only from western Thailand (Kanchanaburi and Tak provinces), presents multiple plausible hypotheses: it may represent a relic population from an ancient western refugium, a cryptic evolutionary lineage with specialized ecological requirements, or a more widespread lineage that appears rare due to limited sampling in western and central regions. The sympatric occurrence of this haplogroup with Haplogroup A without intermediate haplotypes suggests long-term reproductive isolation, though the true geographic extent of Haplogroup D remains uncertain and warrants expanded sampling, particularly in areas east of current western localities. The differential responses of haplogroups to historical processes, with Haplogroup A achieving wide distribution through successful dispersal, Haplogroup B remaining regionally restricted, Haplogroup C isolated in the south, and Haplogroup D appearing as a rare western lineage (potentially due to sampling gaps), were shaped by interacting factors including river capture events, Pleistocene glacial-interglacial cycles creating temporary freshwater corridors [18], drainage reorganization during Asian monsoon intensification (~1 Ma) [51], and subsequent population isolation [47,48]. These patterns mirror those in other Southeast Asian freshwater fishes [46,52], suggesting *T. tambra* experienced biogeographic processes typical of the ichthyofauna of this region, with outcomes determined by species-specific dispersal capabilities and ecological requirements.

While our mitochondrial data reveal distinct phylogeographic patterns, these findings should be interpreted with appropriate caution. Mitochondrial DNA is sensitive to maternal inheritance, introgression, and historical demographic events such as river capture and population bottlenecks, which may not reflect genome-wide patterns. Therefore, definitive validation of these lineages as evolutionary significant units will require complementary analyses using nuclear genome markers (e.g., SNPs, microsatellites) to confirm reproductive isolation and assess bi-parental gene flow patterns. Additionally, sample sizes from certain localities, particularly in southern Thailand, were limited, constraining comprehensive characterization of regional genetic diversity. Future studies with expanded geographic sampling and nuclear markers will be essential to fully resolve population structure across underrepresented areas.

### 4.4. Conservation Implications

The identification of distinct genetic lineages within *T. tambra* populations across Thailand has critical implications for conservation planning, particularly given the ongoing threats to freshwater biodiversity in Southeast Asia [53]. Our findings suggest the need for management approaches that recognize these populations as distinct evolutionary significant units [54], with conservation priorities ranking as follows: (1) highest priority for the genetically unique and geographically restricted Haplogroup D in western Thailand, given its limited distribution and small documented population; (2) high priority for the regionally endemic Haplogroup B confined to northern drainages; and (3) continued monitoring and protection of the widespread Haplogroup A and southern Haplogroup C to maintain their genetic diversity and population connectivity. This approach aligns with successful conservation strategies implemented for other threatened mahseer species in Asia, where recognition of distinct genetic lineages has been crucial for effective conservation planning [19,55,56].

The co-occurrence of different *T. tambra* lineages in northern and western river systems further emphasizes the importance of preserving both genetic diversity and habitat connectivity. Given that *Tor* species require diverse habitat types throughout their life cycle and undertake local migrations for spawning [57,58,59], watershed-based conservation approaches are essential. We recommend implementing integrated conservation strategies that combine the protection of genetically distinct populations with the maintenance of river connectivity, following evidence-based management practices that have proven successful in conserving other threatened freshwater fishes in the region [60].

## 5. Conclusions

This phylogeographic assessment reveals four genetically distinct lineages within Thai *Tor tambra*, demonstrating how the Isthmus of Kra and historical river dynamics have shaped population divergence across major watersheds. Our molecular evidence resolves taxonomic confusion by confirming that specimens from Malaysia and Vietnam, previously assigned to different species, represent misidentified *T. tambra*. Despite limited sampling from certain regions, our findings establish a critical foundation for conserving this economically important species. Protecting these distinct lineages through watershed-based management strategies is imperative for sustaining both wild populations and aquaculture development. As anthropogenic pressures intensify across Southeast Asian freshwater ecosystems, integrating phylogeographic knowledge into conservation planning becomes essential for maintaining the evolutionary potential and long-term viability of mahseer populations throughout the region.

## Figures and Tables

**Table 1:** *Tor tambra* samples used in concatenated analyses (N = 50). All specimens were successfully sequenced for both COI and Cyt*b* markers.

Locality Code	Sampling Sites	COORDINATES	Number of Samples	COI Haplotype Present (Number)	Cyt*b* Haplotype Present (Number)	COI + Cyt*b* Haplotype Present (Number)
Northern Thailand						
N1	Salween River, Mae Hong Son province	17°59′19.0″ N 97°44′10.1″ E	12	A1(1), A3(1), A4(1), B1(5), B2(1), B3(2), B4(1)	A3(1), A4(1), A5(1), B1(8), B2(1)	A2(2), A3(1), A5(1), B1(4), B2(1), B3(2), B4(1),
N2	Nan River, Nan province	18°32′21.4″ N 100°45′55.0″ E	6	A1(5), A2(1)	A2(1), A3(1), B1(4)	A2(2), A6(1), B5(3)
Western Thailand						
W3	Moei River, Tak province	17°23′27.3″ N 98°05′41.9″ E	14	A1(12), A4(1) D1(1)	A1(9), A3(2), A4(1), D1(1)	A1(10), A2(2), A3(1), D1(1)
W4	Tha Song Yang, Tak province	17°26′30.8″ N 98°03′21.8″ E	4	A1(3), A4(1)	A1(3), A4(1)	A1(3), A3(1)
W5	Vajiralongkorn Dam, Kanchanaburi Province	14°47′41.5″ N 98°36′06.8″ E	3	A1(2), D1(1)	A2(2), D1(1)	A5(2), D1(1)
Southern Thailand						
S6	Pran Buri River, Prachuap Khiri Khan province	12°23′22.3″ N 99°43′31.4″ E	1	C3(1)	C3(1)	C3(1)
S7	Khao Suk, Surat Thani province	8°55′16.6″ N 98°50′20.2″ E	1	C2(1)	C2(1)	C4(1)
S8	Banglang, Yala province	6°08′02.8″ N 101°17′57.1″ E	6	C1(5), C4(1)	C1(4), C4(2)	C1(3), C2(1), C5(2)
S9	Bala, Narathiwat province	5°48′51.9″ N 101°51′55.2″ E	3	C1(3)	C1(3)	C1(3)
Total			50	50	50	50

**Table 2:** Pairwise genetic distances (K2P, %) among *Tor tambra* clades.

	Clade A	Clade B	Clade C	Clade D
Clade A	—	1.69	1.91	2.17
		1.55–2.04	1.65–2.44	2.04–2.44
Clade B	**2.16**	—	1.55 *	1.65
	2.01–2.38		1.45–1.74	1.65–1.74
Clade C	1.52	1.50 *	—	**2.24 ^†^**
	1.27–2.01	1.27–1.82		2.14–2.34
Clade D	2.22	**2.50 ^†^**	1.91	—
	2.19–2.38	2.38–2.57	1.64–2.19	

Lower triangle: COI (672 bp); Upper triangle: Cyt*b* (1140 bp). Values shown as mean (first line) and range (second line). Bold values marked with * indicate the lowest interclade divergence; bold values marked with **^†^** indicate the highest interclade divergence for the respective marker.

## Data Availability

The original contributions presented in this study are included in the article/Supplementary Material. Further inquiries can be directed to the corresponding authors.

## Supplementary Materials

The following supporting information can be downloaded at https://www.mdpi.com/article/10.3390/ani16030517/s1. Figure S1: Maximum likelihood (ML) phylogenetic tree depicting the relationship among *Tor tambra* and closely related species, inferred from mitochondrial COI sequences.; Figure S2: Maximum likelihood (ML) phylogenetic tree depicting the relationship among *Tor tambra* and closely related species, inferred from mitochondrial Cyt*b* sequences; Table S1: Detail of specimens for *Tor* and related genera used in the study; Table S2: The best partitioning scheme and models of ML and BI methods for all datasets; Table S3: Kimura 2-parameter (K2P) genetic distances for COI sequences; Table S4: Kimura 2-parameter (K2P) genetic distances for Cyt*b* sequences; Table S5: Kimura 2-parameter (K2P) genetic distances for 136 COI sequences of *Tor* and *Neolissochilus* species.
