# Supplementary material for: Phylogeographic Structure Reveals Hidden Diversity Patterns in Tor tambra (Cyprinidae) Across Thai River Systems
Source: Animals (Basel). 2026 Feb 6;16(3):517. doi: 10.3390/ani16030517 (PMC12897184; doi:10.3390/ani16030517)
Supplement: Supplementary file 1 [file animals-16-00517-s001.zip › Figures S1-S2.pdf]

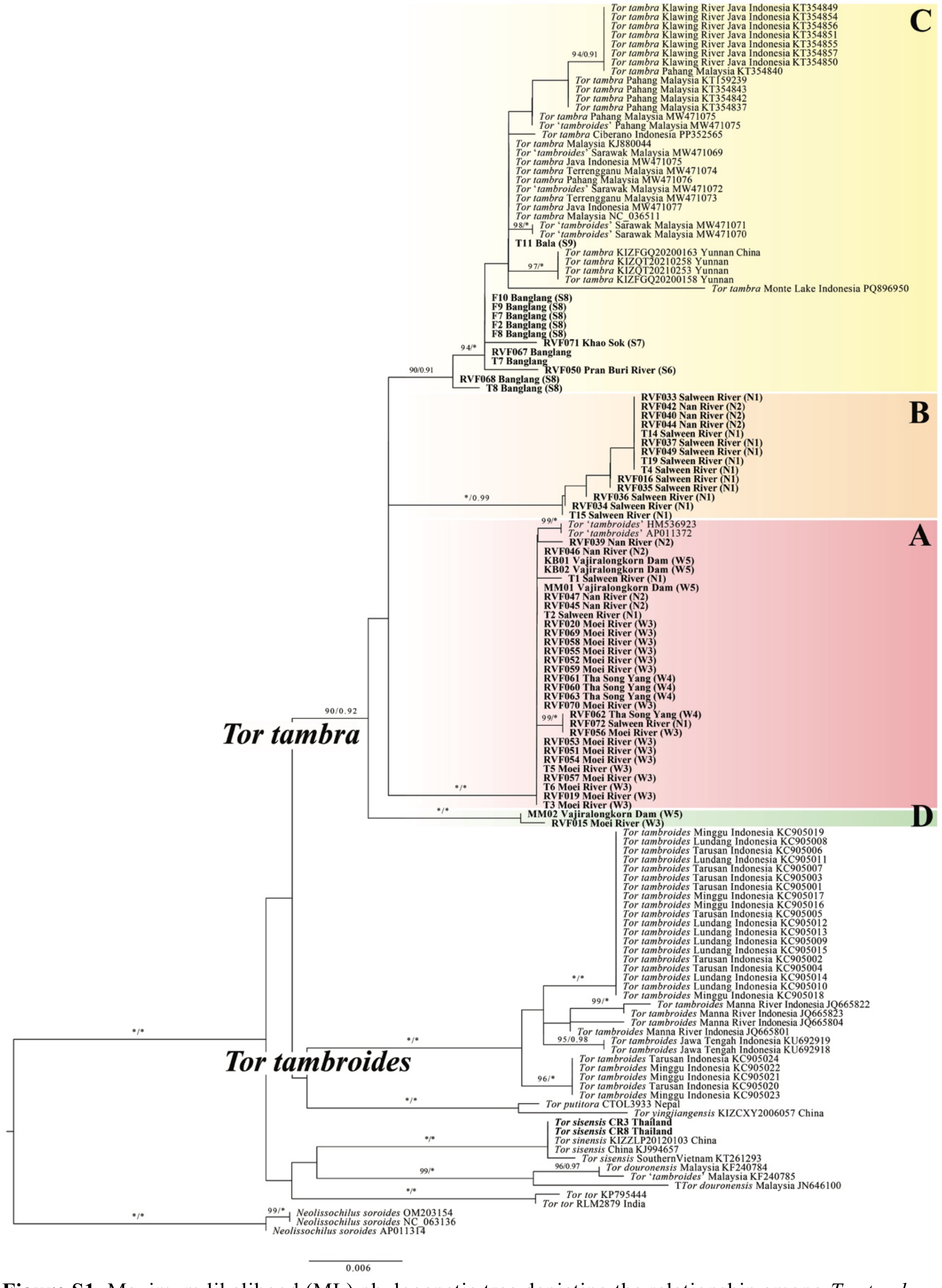

Figure S1. Maximum likelihood (ML) phylogenetic tree depicting the relationship among *Tor tambra* and closely related species, inferred from mitochondrial COI sequences. Sequences generated in this study are highlighted in bold. Support values from ML bootstrap analysis (left) and Bayesian posterior probabilities (right) are displayed at the nodes. An asterisk (*) denotes full support (100%, 1.0) in both analyses. Only bootstrap values greater than 90% and posterior probabilities exceeding 0.9 are presented.

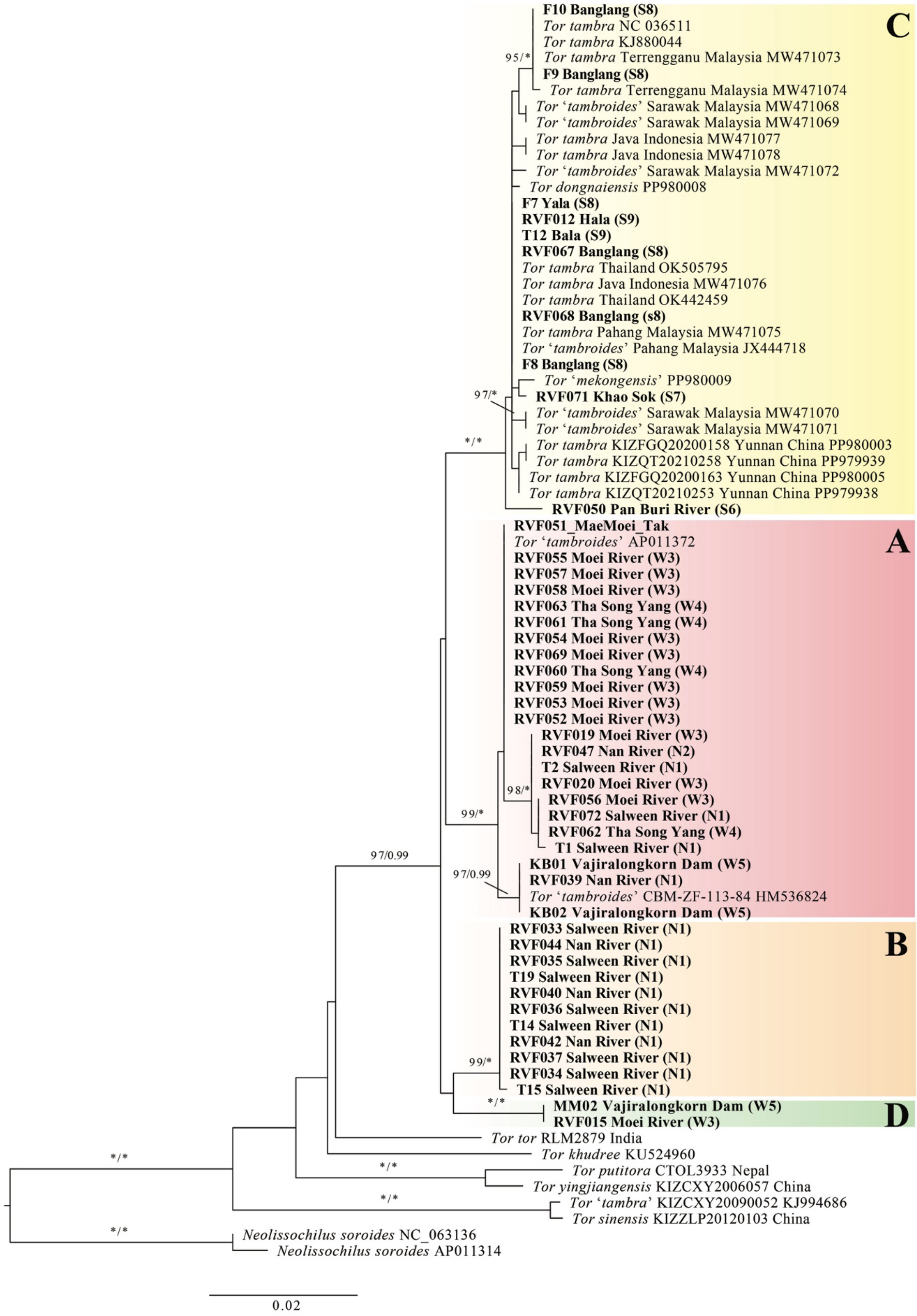

Figure S2. Maximum likelihood (ML) phylogenetic tree depicting the relationship among *Tor tambra* and closely related species, inferred from mitochondrial Cyt*b* sequences. Sequences generated in this study are highlighted in bold. Support values from ML bootstrap analysis (left) and Bayesian posterior probabilities (right) are displayed at the nodes. An asterisk (*) denotes full support (100%, 1.0) in both analyses. Only bootstrap values greater than 90% and posterior probabilities exceeding 0.9 are presented.
